# Systems Biological Analysis of Immune-Metabolic Host Responses to Distinct Malaria Parasites

**DOI:** 10.1021/acsomega.5c07785

**Published:** 2025-10-13

**Authors:** Davi Vinícius Lima, Tiago Paiva Guimarães, Anne Cristine Gomes Almeida, Arthur Jesuz Teixeira, Wuelton Marcelo Monteiro, Gisely Cardoso Melo, Luiz Gustavo Gardinassi

**Affiliations:** † Programa de Pós-Graduação em Medicina Tropical e Saúde Pública, Instituto de Patologia Tropical e Saúde Pública, Universidade Federal de Goiás, 74605-050 Goiânia, Goiás, Brazil; ‡ Gerência de Malária, 200241 Fundação de Medicina Tropical Doutor Heitor Vieira Dourado, 69040-000 Manaus, Amazonas, Brazil; § Departamento de Enfermagem Materno-Infantil e Saúde Pública, Escola de Enfermagem de Ribeirão Preto, 28133 Universidade de São Paulo, 14040-902 Ribeirão Preto, São Paulo, Brazil; ∥ Escola Superior de Ciências da Saúde, 468981 Universidade do Estado do Amazonas, 69065-001 Manaus, Amazonas, Brazil

## Abstract

Recent studies suggest differential activation of immune cells and biochemical pathways during infection with distinct etiological agents of human malaria, *Plasmodium falciparum* or *Plasmodium vivax*. Rhesus monkeys infected with *Plasmodium coatneyi* develop pathology comparable to *P. falciparum* infection in humans, whereas *P. vivax* is modeled by infection with *Plasmodium cynomolgi*. To investigate host immune and metabolic responses, we analyzed and integrated public blood flow cytometry, transcriptomics, and untargeted metabolomics data from simian infection models and human malaria. We found conserved dynamics of blood dendritic cells, effector memory CD8+ T cells, and PD-1+ effector memory CD8+ T cells in simian infections; increased expression of *HMOX1* and coagulation-related genes was conserved in simian infections, while the complement activation gene module was upregulated in rhesus and humans. Myeloid cell transcriptional responses were enriched in all infections, but gene modules reflecting chemokine responses and T cell differentiation were mostly associated with *P. cynomolgi* and *P. vivax*. Untargeted metabolomics analysis suggests conserved regulation of metabolites such as kynurenine and androgen- and estrogen-derived metabolites, and metabolic modules indicate tyrosine metabolism activity. Multimodal data integration from rhesus monkeys revealed distinct interacting network models. In conclusion, we identified conserved cellular, transcriptional, and metabolic responses between simian models and human malaria. Moreover, many significant variables were associated with one determined *Plasmodium* species, suggesting a significant impact of parasite biology on immune and metabolic host responses. However, systematic experimental comparisons are needed to distinguish species-specific host responses to *Plasmodium* from those that are caused by factors beyond the infection.

## Graphical abstract

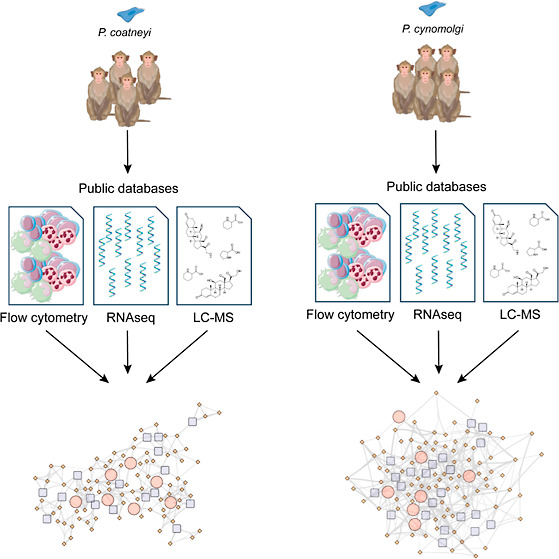

## Introduction

Malaria remains one of the most challenging global threats caused by infectious microbes. *Plasmodium falciparum* is the most lethal etiological agent, but *Plasmodium vivax* underlies most cases of malaria outside sub-Saharan Africa. There are fundamental aspects of biology from these phylogenetically distant parasites that promote unique pathology and clinical outcomes depending on the species. *P. vivax* has a tropism for reticulocytes, which compose a small fraction of circulating red blood cells (RBCs),^1,2^ while its major biomass is thought to localize in hematopoietic niches such as spleen^3^ and bone marrow.^4,5^ Furthermore, *P. vivax* can form hypnozoites, a dormant liver stage capable of reactivating weeks or months after the primary infection, leading to a new malaria episode. In contrast, *P. falciparum* invades immature and mature RBCs and often reaches higher levels of peripheral parasitemia, but the total biomass also includes infected RBCs sequestered in capillaries from several organs.^6^ *P. vivax* gametocytes appear earlier than *P. falciparum* during the blood-stage infection and enhance its transmission. Therefore, distinct *Plasmodium* species evolved alternative strategies to persist. The impact of such differences on host immunity and metabolism is not completely understood.

The host defense against *Plasmodium* infection is complex and engages a myriad of cells via cytokines, chemokines, and bioactive metabolites. The immunological mechanisms that protect and promote pathology have been studied in humans and animal models of malaria,^7,8^ but few studies compared host responses to malaria caused by distinct species. *P. vivax* elicits fever at lower parasite densities,^9^ which can explain higher inflammatory responses and endothelial activation compared to *P. falciparum*.^10–12^ Infection with *P. falciparum* induces higher activation of CD4^+^ T lymphocytes and regulatory T cells.^10^ Studies using mice infected with different *Plasmodium* demonstrated heterogeneous kinetics of parasitological, clinical, and transcriptional features that depend on the parasite species and strain.^13^ However, because of the evolutionary distance between humans and mice, better models are required to understand immune and metabolic responses in humans, especially to *P. vivax* infection.

Controlled infections of *Macaca mulatta* (rhesus macaque) with *Plasmodium coatneyi* or *Plasmodium cynomolgi* are relevant models of human malaria caused by *P. falciparum* ^14,15^ or *P. vivax*,^16^ respectively. Systems immunology approaches were applied to study *P. coatneyi* or *P. cynomolgi* infections individually, providing important insights into simian *Plasmodium* infection and malaria pathogenesis.^17–19^ In principle, the data sets acquired in these studies were not meant to be compared, because they were obtained at different periods of time, using different animals and other circumstances. But controlled infections were performed in the same environment, and the data were acquired with instruments at the same institutions. Therefore, we explored the data sets to understand whether they could be used to investigate the impact of *Plasmodium* species on the host immune response and metabolism. Performing a careful examination of the experimental design between the controlled infections with *P. coatneyi* or *P. cynomolgi*, we analyzed, integrated, and compared immune cell phenotyping, transcriptomics, and metabolomics data. We also gathered further translational insights by comparing them with data from humans with malaria caused by *P. falciparum* or *P. vivax*. Our study demonstrates that rhesus and humans exhibit conserved immune and metabolic responses to distinct malaria parasites. Linking specific host features to a determined *Plasmodium* species is challenging because of the diverse confounding effects that emerge from different experimental settings. However, multimodal network models suggest heterogeneous activation and communication between cellular and biochemical responses to distinct malaria parasites.

## Results

### Different *Plasmodium* Species Induce Similar Cell Blood Counts at Peak Parasitemia

To investigate how host responses are influenced by infection with different *Plasmodium* species, we leveraged two public large-scale multimodal data sets acquired from rhesus macaques (*M. mulatta*) submitted to controlled infection with the *P. coatneyi* Hackeri strain^17^ or the *P. cynomolgi* B strain^20^ (Table 1).

**Table 1:** Summary Information about the Data Sets and Experimental Settings^a^

*Plasmodium*	*P. coatneyi* ^17^	*P. cynomolgi* ^20^
sex	male	male
age	∼4 years	∼3 years
weight	8.0–9.0 kg	5.3–6.3 kg
sample size	4	5
animal ID	RUn13, RTi13, RZe13, RWr13	RFa14, RIc14, RFv13, RMe14, RSb14
infection date	Mar/2014	Sep/2013
inoculum	100 sporozoites	2000 sporozoites
MaHPIC Exp	E03	E04

a MaHPIC Exp: Malaria Host–Pathogen Interaction Center Experiment. All data are available at PlasmoDB (https://plasmodb.org/plasmo/app/static-content/PlasmoDB/mahpic.html).

Blood samples were collected before infection (baseline), at the peak of parasitemia (acute infection), and at other time points by the Malaria Host–Pathogen Interaction Center (MaHPIC) consortium.^20^ Parasitemia peaked on day 22 postinfection with *P. coatneyi* and on days 19–21 postinfection with *P. cynomolgi*. Importantly, there were no significant differences in parasitemia during acute infection between experiments (Figure 1A). White blood cell (WBC), red blood cell (RBC), and platelet counts diminished at peak parasitemia compared to baseline (Figure 1E–J), but there were no significant differences in cell blood counts between infections with distinct *Plasmodium* (Figure 1B–D). There are clear particularities in parasite biology, as *P. coatneyi* reached similar parasite densities in the peripheral blood, despite the initial parasite inoculum differing by 20 times, with an estimated 100 sporozoites for *P. coatneyi* ^17^ and 2000 sporozoites for *P. cynomolgi*.^20^ In contrast, patterns of cell blood counts suggest conserved cellular responses during malaria caused by different *Plasmodium* species.

**Figure 1:**
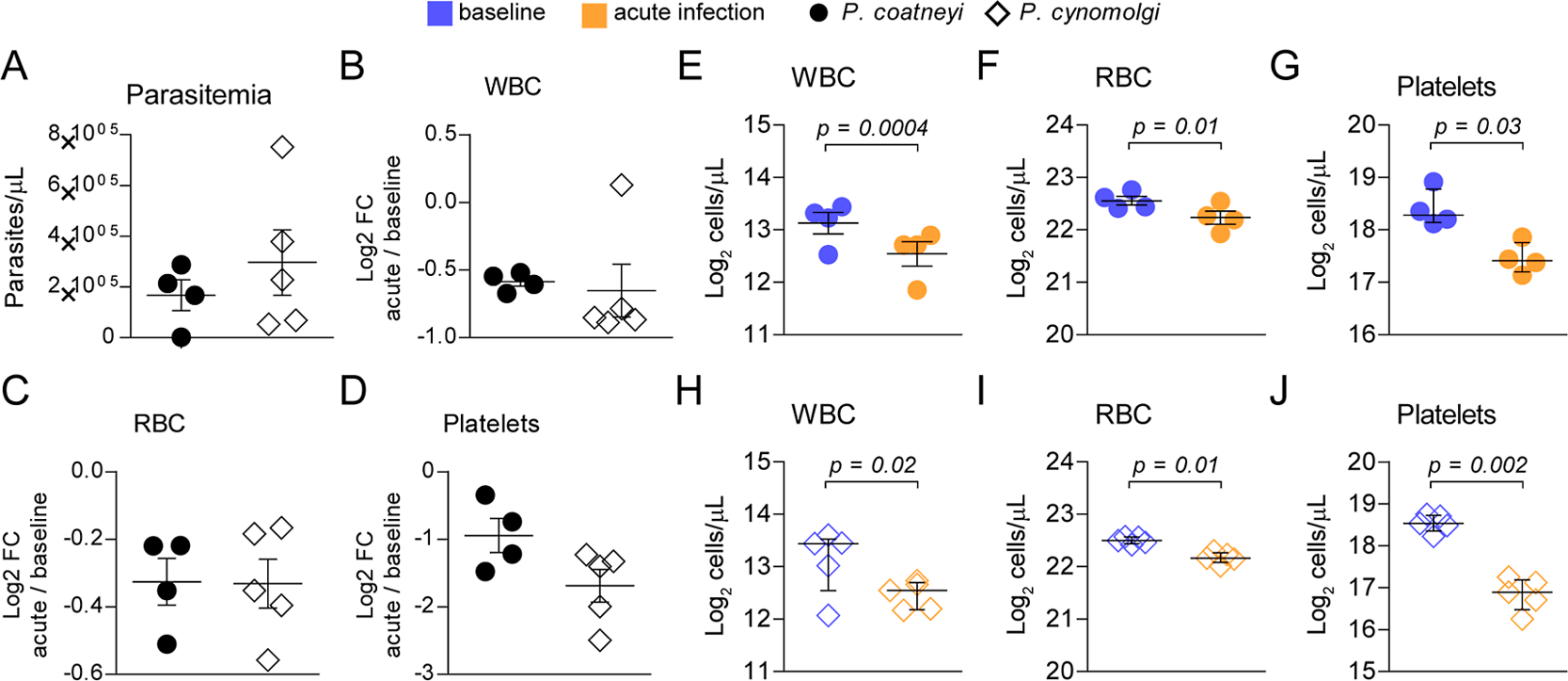
Parasitemia and cell blood counts of rhesus macaques infected with *P. coatneyi* or *P. cynomolgi*. (A) Number of parasites at the peak of infection (acute infection). (B) White blood cell (WBC) counts, (C) red blood cell (RBC) counts, and (D) platelet counts at acute infection normalized by individual baseline levels. (E) WBC, (F) RBC, and (G) platelet counts at baseline and acute infection with *P. coatneyi*. (H) WBC, (I) RBC, and (J) platelet counts at baseline and acute infection with *P. cynomolgi*. Statistical analyses comparing different experiments (A–D) were performed with the Mann–Whitney test; comparisons of different time points (E–J) were analyzed with the paired *t*-test.

### Immune Cell Profiles in the Peripheral Blood Are Impacted by *Plasmodium* Species

We analyzed the frequency and absolute numbers of innate and adaptive immune cells as determined previously by flow cytometry.^20^ Compared to the baseline, *P. coatneyi* was associated with a higher frequency of CD4^+^ and CD8^+^ T cells expressing the proliferation marker Ki67 (Figure 2A). The frequency of tissue-like memory B and caspase-3^+^ B cells also increased, but dendritic cells (DCs) decreased. In agreement, the numbers of DCs decreased and Ki67^+^ central memory (CM) CD4^+^ T cells increased, but the numbers of other subsets of monocytes, DCs, and CD8^+^ T cells reduced (Figure 2B). The expression of markers for myeloid cell activation, such as CD83 and HLA-DR, was reduced in DCs (Figure 2C), whereas Ki67^+^ central memory (CM) CD4^+^ T cells displayed reduced cell death markers such as caspase-3 and CD95 (Fas) but increased CD28 and PD-1 expression (Figure 2D).

**Figure 2:**
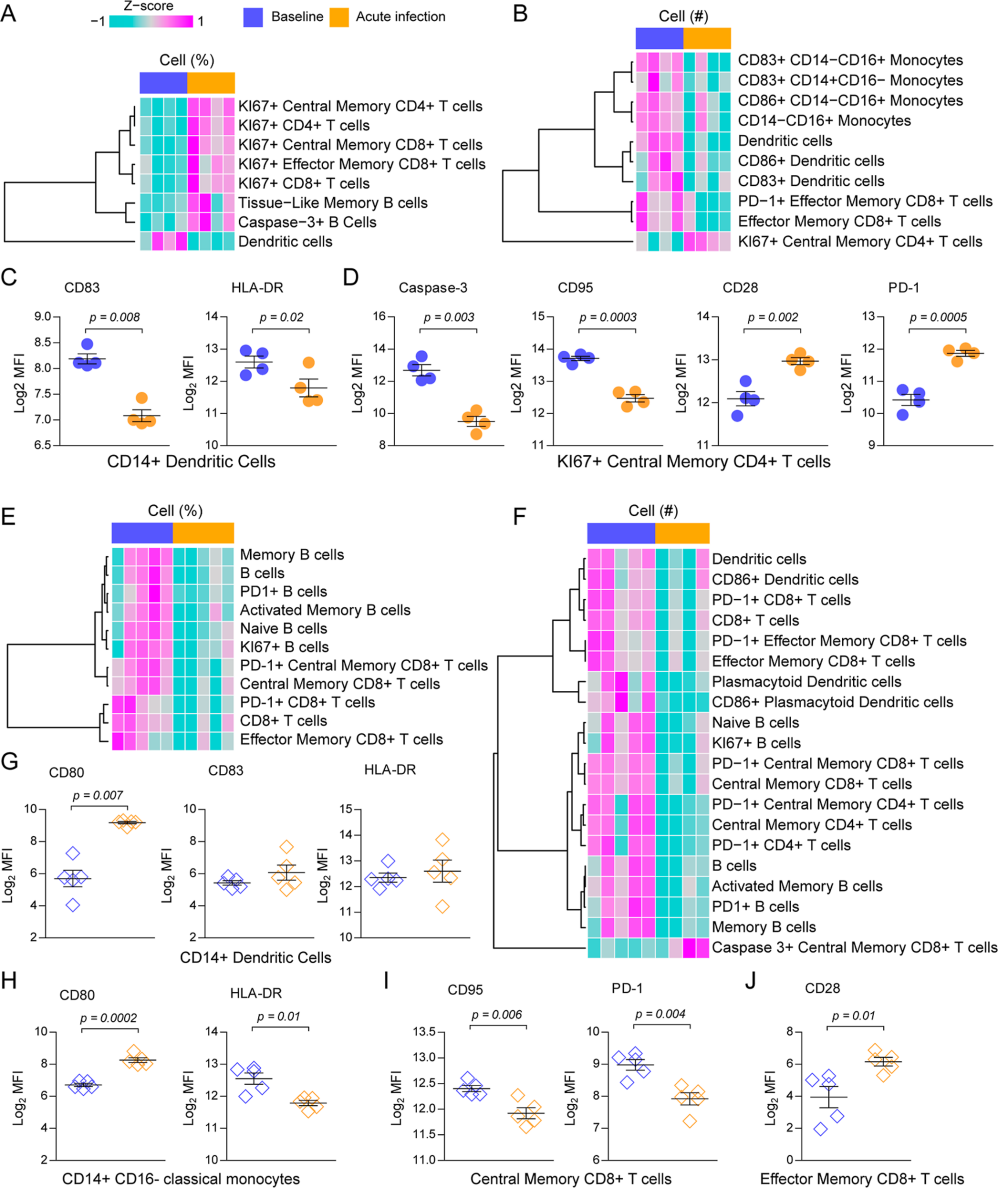
Blood immune cell profiling of rhesus macaques infected with *P. coatneyi* or *P. cynomolgi*. Innate and adaptive immune cell phenotyping was performed with flow cytometry. (A) Cell frequencies and (B) absolute cell numbers differing from baseline levels at acute infection with *P. coatneyi*. (C) Log2-transformed median fluorescence intensity (MFI) of CD83 and HLA-DR in myeloid, CD14^+^ dendritic cells. (D) Log2 MFI of caspase-3, CD95, CD28, and PD-1 in Ki67+ central memory CD4+ T cells. (E) Cell frequencies and (F) absolute cell numbers differing from baseline levels at acute infection with *P. cynomolgi*. (G) Log2 MFI of CD80, CD83, and HLA-DR in myeloid, CD14^+^ dendritic cells. (H) Log2 MFI of CD80 and HLA-DR in CD14^+^CD16^–^ classical monocytes. (I) Log2 MFI of CD95 and PD-1 in central memory CD8+ T cells. (J) Log2 MFI of CD28 in effector memory CD8+ T cells.

Compared to the baseline, infection with *P. cynomolgi* reduced the frequency of diverse B and CD8+ T cell subsets (Figure 2E), but there were no overlaps with subsets differing on *P. coatneyi* infection (Figure 2A). There were reduced numbers of DCs, CD86^+^ DCs, effector memory (EM) CD8^+^ T cells, and PD-1^+^ EM CD8^+^ T cells in both infections compared to those in the baseline (Figure 2F). *P. cynomolgi* also reduced the numbers of plasmacytoid DC (pDC), B cell, CD4^+^ T cell, and CD8^+^ T cell subsets while increasing caspase-3^+^ CM CD8^+^ T cells (Figure 2F). Despite being reduced in numbers, DCs increased the expression of CD80 but not CD83 or HLA-DR (Figure 2G). CD14^+^ CD16^–^ classical monocytes also increased CD80 but expressed lower levels of HLA-DR (Figure 2H). Among T cell subsets, CM CD8^+^ T cells expressed lower levels of CD95 and PD-1 (Figure 2I), whereas EM CD8^+^ T cells increased CD28 expression (Figure 2J).

### Conserved and Specific Transcriptional Host Responses to Distinct *Plasmodium*

We observed substantial differences in the dynamics of immune cell composition and phenotypes in the peripheral blood of rhesus macaques infected with *P. coatneyi* or *P. cynomolgi*. These differences suggest that distinct molecular programs coordinate the functions of such subsets. To gather further insights into the host response, we analyzed blood transcriptomes at the same time points. We analyzed 11,279 and 12,379 genes for infection with *P. coatneyi* and *P. cynomolgi*, respectively. We found 495 differentially expressed genes (DEGs) in *P. coatneyi* and 488 DEGs in *P. cynomolgi* infection with false discovery rate (FDR) adjusted *p* < 0.05 and log fold-change > 1 or < −1 (Figure 3A). *CTLA4* was the most significantly expressed gene in *P. coatneyi* infection, a gene that composes a transcriptional biomarker signature of *P. falciparum* infection.^21^ *CTLA4* was also significantly expressed in *P. cynomolgi* infection, but *PPBP (CXCL7)* was among the most significant, suggesting the activation of platelets and blood coagulation. There were 239 common DEGs between the infections (Figure 3B), and as expected, the level of *HMOX1* expression increased in both infections (Figure 3C). This gene encodes the enzyme heme oxygenase-1, which is critical to protect from oxidative effects of heme released upon RBC rupture.^22^ Overrepresentation analysis of gene ontologies revealed conserved responses related to interactions between organisms, defense response, regulation of immune response, inflammatory response, and blood coagulation and hemostasis (Figure S1A). Baseline-subtracted expression of genes related to blood coagulation and hemostasis was almost identical between different infections (Figure S1B).

**Figure 3:**
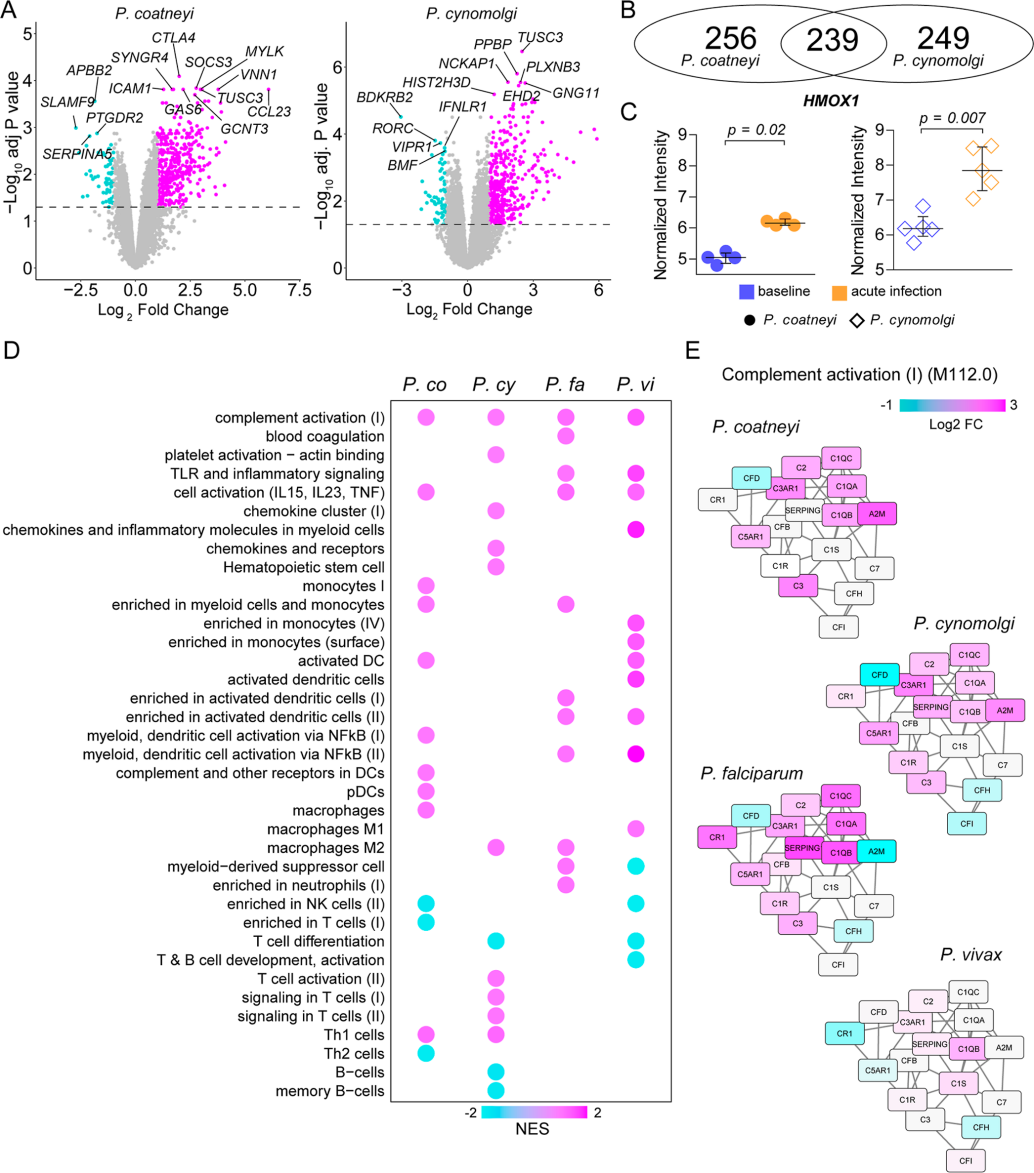
Transcriptomic analyses of host blood from infections with *P. coatneyi*, *P. cynomolgi*, *P. falciparum*, and *P. vivax*. (A) Differentially expressed genes (DEGs) in blood of rhesus macaques between baseline and acute infection with *P. coatneyi* or *P. cynomolgi*. (B) Overlapping DEGs during acute infection with *P. coatneyi* or *P. cynomolgi*. (C) Normalized expression of *HMOX1* at baseline and acute infection with *P. coatneyi* or *P. cynomolgi*. (D) Gene set enrichment analyses using the Blood Transcription Modules framework of simian and human *Plasmodium* infections. (E) Representative networks of genes from BTM M112.0 Complement activation (I) at acute infection with *P. coatneyi*, *P. cynomolgi*, *P. falciparum*, and *P. vivax*. NES: normalized enrichment score.

DEGs associated specifically with *P. coatneyi* infection are enriched in differentiation, development, myeloid cell homeostasis, erythrocytes, and metabolism of reactive oxygen species. DEGs induced solely by *P. cynomolgi* are enriched in inflammatory response, cytokine production, regulation of defense response, cell activation, and interaction between organisms (Figure S1C). The expression of individual genes can be influenced by differences in experiments, animals, and parasite inoculum, among many others. Therefore, we used a modular approach that also allowed us to compare with transcriptional responses in whole blood of children infected with *P. falciparum* (PRJEB45911) or *P. vivax* (GSE144792). We performed gene set enrichment analysis (GSEA) using blood transcription modules (BTM)^23^ as gene sets (Figure 3D). We identified the upregulated activity of genes involved in complement activation across all infections (Figure 3E). Except for *P. cynomolgi*, the module reflecting cell activation by IL-15, IL-23, and TNF was also conserved among rhesus and humans. Transcriptional activity of Th1 cells was conserved in *P. coatneyi* and *P. cynomolgi*. Furthermore, we found a myeloid cell and monocyte module to be upregulated in both *P. coatneyi* and *P. falciparum* infections, whereas *P. cynomolgi* and *P. vivax* were both associated with modules related to chemokines, chemokine receptors, and inflammatory molecules in myeloid cells. Indeed, all infections were associated with myeloid cell activity, but the regulation of myeloid-derived suppressor cell activity differed between *P. falciparum* and *P. vivax* (Figure 3D).

We also identified many modules associated with specific *Plasmodium* species. For example, *P. coatneyi* was associated with diverse modules reflecting cell division (Figure S2), which corroborates proliferating CD4^+^ T cells observed in flow cytometry (Figure 2A). Among modules associated with *P. cynomolgi*, the reduced activity of B cells (Figure 3D) also supports flow cytometry data (Figure 2E,F). Moreover, *P. cynomolgi* was also associated with integrins, extracellular matrix, and glycerophospholipid metabolism (Figure S2). The results demonstrate many conserved transcriptional responses to *Plasmodium* irrespective of the species in rhesus and humans. However, infections by different malaria parasites promote heterogeneous transcriptional programs in general.

### Malaria Induces Conserved Regulation of Immunomodulatory Metabolites

The inflammatory response to *Plasmodium* infection is tightly associated with the metabolic activity of immune cells^24^ and metabolites circulating systemically.^25,26^ We compared untargeted metabolomics data acquired with liquid chromatography–mass spectrometry (LC–MS) from plasma of the same animals and from humans infected with *P. falciparum* or *P. vivax* (MTBLS664). We analyzed thousands of *m*/*z* peaks at positive and negative ionization modes detected in plasma from rhesus and humans. Among the annotated metabolites, kynurenine increased after both simian infections (Figure 4A). This increase in kynurenine has been well documented in humans infected with *P. falciparum* ^17^ and *P. vivax*.^25^ Moreover, we found increased *SLC7A5* (Figure 4B) and *IDO1* (Figure 4C) expression in rhesus and human blood, suggesting that peripheral leukocytes catabolize tryptophan into kynurenine during infection.

**Figure 4:**
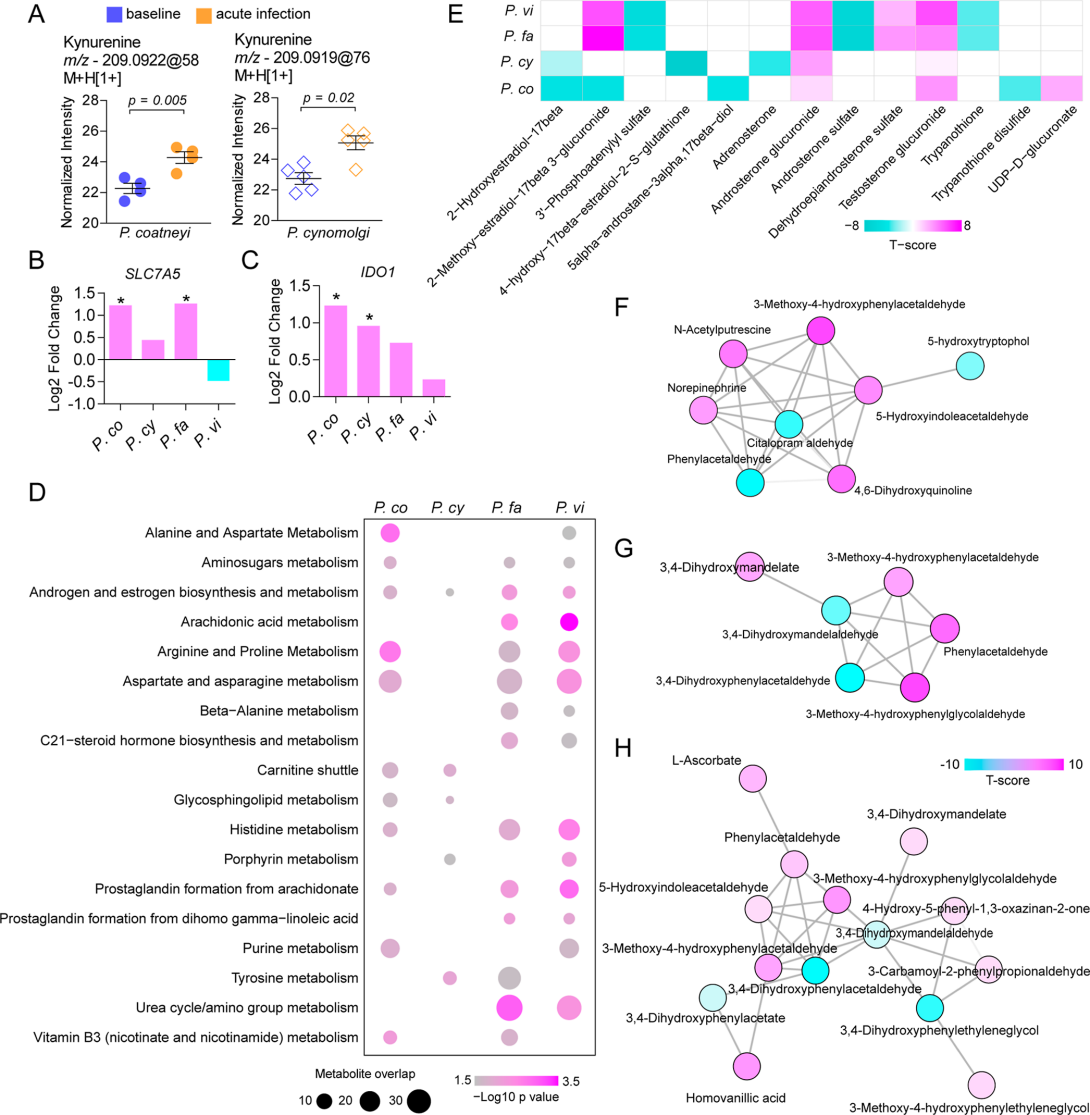
Plasma metabolomic phenotypes from rhesus macaques infected with *P. coatneyi* or *P. cynomolgi*. (A) Normalized abundance of kynurenine at baseline and acute infection with *P. coatneyi* or *P. cynomolgi*. (B,C) Log2 fold change of *SLC7A5* (B) and *IDO1* (C) in infected rhesus compared to baseline or infected humans compared to uninfected controls. (D) Metabolic pathway prediction analysis in rhesus and humans infected with different *Plasmodium*. (E) Annotated metabolite features in the androgen and estrogen biosynthesis and metabolism pathway. (F–H) Predicted metabolic modules overlapping with the tyrosine metabolism pathway in *P. coatneyi* (F), *P. falciparum* (G), and *P. vivax* (H) infections. Each connection represents a known reaction.

We used the mummichog software to evaluate biological enrichment of *m*/*z* features, which is specifically designed to predict metabolic activity from untargeted metabolomics data.^27^ We also compared this with untargeted metabolomics data from humans with malaria caused by *P. falciparum* or *P. vivax*. Many of the predicted pathways were enriched in both rhesus and humans, with a few exceptions (Figure 4D). We observed that vitamin B3 metabolism was enriched for *P. coatneyi* and *P. falciparum*, whereas porphyrin metabolism was enriched for *P. cynomolgi* and *P. vivax*, suggesting that parasite biology might affect metabolites related to these pathways. The androgen and estrogen biosynthesis and metabolism pathway was enriched for all infections, with most metabolites displaying conserved regulation. The analysis suggests, for example, an increased abundance of androsterone glucuronide and testosterone glucuronide (Figure 4E). In addition to pathway analysis, we also analyzed metabolic modules, which represent networks of known reactions predicted from the data. By comparing significant modules from rhesus and human infections, we identified modules which overlap with the tyrosine metabolism pathway in *P. coatneyi* (Figure 4F), *P. falciparum* (Figure 4G), and *P. vivax* (Figure 4H). A detailed analysis demonstrates that the reaction between 3-methoxy-4-hydroxyphenylacetaldehyde and phenylacetaldehyde is present in the three modules.

There were species-specific pathways enriched in the data sets, such as glycerophospholipid metabolism for *P. cynomolgi* (Figure S3A), which was also specifically associated with this infection in transcriptomic data from whole blood (Figure S2). Annotated metabolites from this pathway suggest an increased abundance of glycerol-phosphocholine (Figure S3B) but reduced abundance of lysoPC (14:0) (Figure S3C) in both simian infections. However, *P. cynomolgi* also increased the level of sphinganine and reduced the abundance of sphingosine-1-phosphate (S1P) (Figure S3D). S1P gradients regulate lymphocyte trafficking from lymphoid tissues to blood,^28^ explaining reduced lymphocyte abundance in the blood during acute *Plasmodium* infection (Figure 2E,F). In addition, the data suggest that the abundance of pyridoxamine phosphate increases in *P. cynomolgi* but not in *P. coatneyi* infection (Figure S3E).

### Integrative Response Networks of Rhesus Macaque Infection with Distinct Malaria Parasites

Considering that the immune and metabolic responses are tightly controlled and regulate each other, we sought to capture the concerted activity of cells, genes, and metabolites upon infection with different *Plasmodium* species. For that, we used a modified hierarchical community method to integrate data types of different natures.^25,29^ First, all data from the peak parasitemia were normalized to baseline values to exclude potential confounding effects for each individual animal. Following, gene matrices were reduced to blood transcription modules (BTMs),^23^ followed by hierarchical clustering to group BTMs based on Pearson correlation. The same hierarchical clustering method was used for the matrices of cell frequencies and *m*/*z* features. Retention time was also considered for metabolite clusters to ensure the grouping of *m*/*z* features related to the same compound. Associations between different data types were assessed at the cluster level using partial least-squares (PLS) correlations, whose significance was tested with permutations.

The integrative network of *P. coatneyi* infection displays 113 nodes linked by 298 edges (Figure 5A). We identified a highly connected subnetwork including cell cluster 2, two BTM clusters, and three metabolite clusters (Figure 5B). Here, each of the nodes of the network is composed of correlated features. For example, cell cluster 2 aggregates correlated B cell subsets, KI67+ central memory CD8+ T cells, γδ T cells, and CD83+ DCs (Figure 5C). BTM cluster 11 is composed of BTMs reflecting T cells (Figure S4A), which is also evident at the gene level (Figure 5D). Predicted activity of metabolite cluster 33 was related to lipids such as prostaglandins, glycosphingolipids, and glycerophospholipids, whereas metabolite clusters 34 and 47 were associated with amino acid metabolism (Figure 5E).

**Figure 5:**
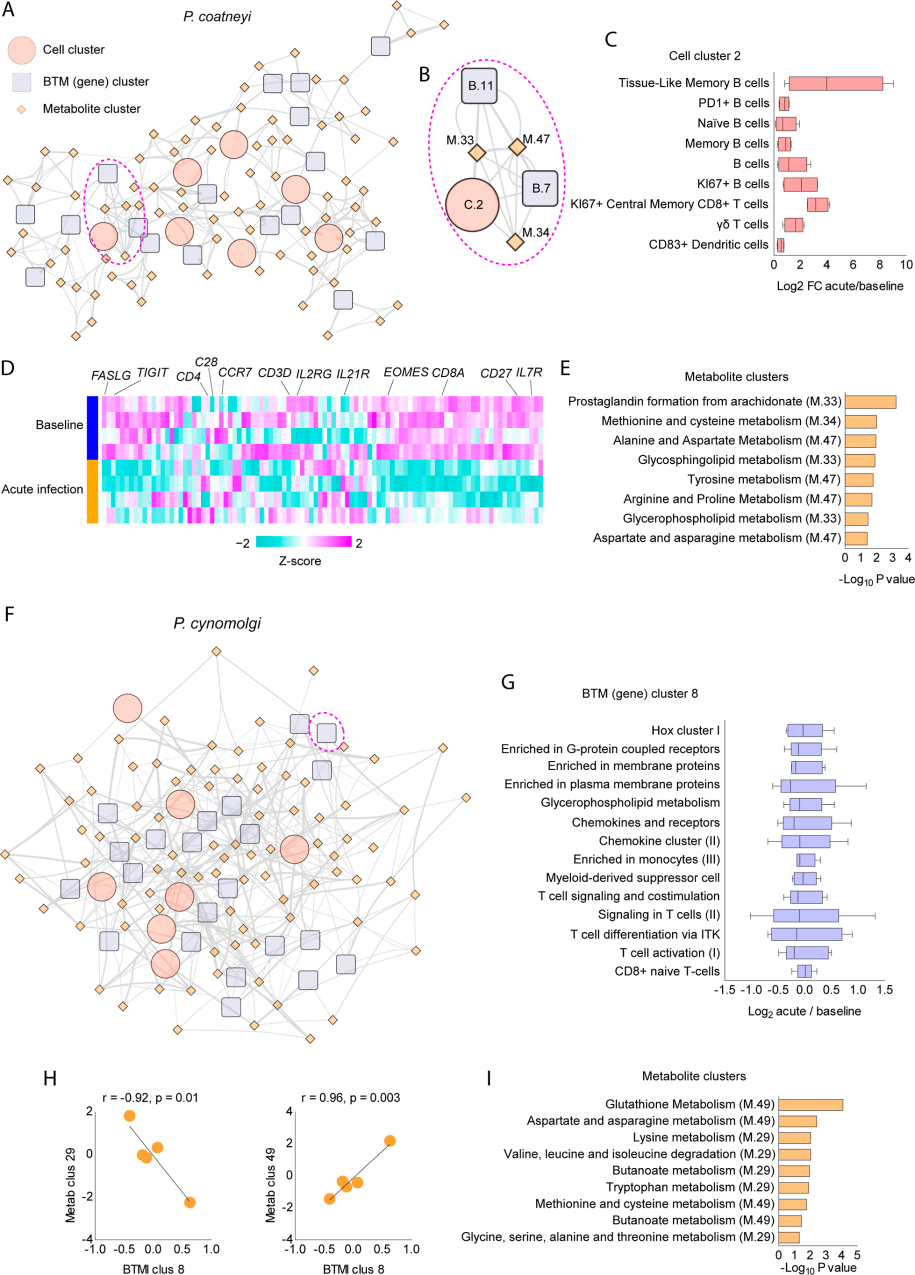
Integrative multimodal analyses of rhesus macaques infected with *P. coatneyi* or *P. cynomolgi*. (A) Multiscale, multifactorial response network (MMRN) of rhesus macaques infected with *P. coatneyi*. (B) Subnetwork of interconnected nodes anchored by cell cluster C.2. (C) Cell subsets are grouped in cell cluster C.2. (D) Normalized expression of genes associated with BTM cluster B.11. (E) Metabolic pathway predictions for metabolite clusters M.33, M.34, and M.47. (F) MMRN rhesus macaques infected with *P. cynomolgi*. (G) BTMs composing BTM cluster B.8. (H) Correlations between BTM cluster B.8 and metabolite clusters M.29 and M.49. (I) Metabolic pathway predictions for metabolite clusters M.29 and M.49.

Integrative analysis of data from rhesus macaques infected with *P. cynomolgi* resulted in 116 nodes linked by 342 edges (Figure 5F). An example is given by BTM cluster 8, which aggregates genes related to G-coupled receptors, membrane receptors, glycerophospholipid metabolism, chemokines, and T cells, among others (Figure 5G). It was negatively correlated with metabolite cluster 29 and positively correlated with metabolite cluster 49 (Figure 5H). Mummichog analysis predicted significant activity for the metabolism of glutathione and many amino acids, while both metabolite clusters were enriched for butanoate metabolism (Figure 5I), demonstrating the redundancy of the data.

## Discussion

In this study, we compared blood immune cell profiles, transcriptional signatures, and metabolomes from rhesus macaques infected with *P. coatneyi* or *P. cynomolgi*. To understand the translational potential from our findings, we also compared the results to transcriptomics and untargeted metabolomics data from humans with malaria caused by *P. falciparum* and *P. vivax*. We identified conserved molecular responses that pinpoint hallmarks of malaria, irrespective of *Plasmodium* species. Cell blood counts were similar between experiments in rhesus, and many overlapping features occurred at the transcriptional layer, including genes that code for critical molecules during malaria, such as heme oxygenase-1.^30^ The expressions of genes encoding coagulation and hemostasis molecules were almost identical, demonstrating highly conserved responses. Importantly, conserved activity of the complement gene module in rhesus and human data sets has also been documented in the bone marrow of *P. vivax* patients,^31^ reflecting classical immunological mechanisms of resistance to *Plasmodium* infection. Compared to the baseline, there is clear leukopenia at the peak of parasitemia in rhesus macaques, but genes in whole blood were mostly upregulated. Most BTMs differed between infections in rhesus and humans, but all of them were associated with at least one module reflecting myeloid cell activity. The gene module enriched in myeloid cells and monocytes was upregulated only in *P. coatneyi* and *P. falciparum* infections, while chemokines and receptors were upregulated and T cell differentiation was downregulated only for *P. cynomolgi* and *P. vivax*. These results suggest that parasite biology may influence how hosts respond to distinct *Plasmodium* and may have implications for vaccine development.

Biochemical analysis of blood from humans and nonhuman models of malaria has been associated with higher abundance of kynurenine due to interferon-mediated tryptophan catabolism via increased expression of indoleamine 2,3-dioxygenase (IDO1).^17,25,32–34^ Expression of *SLC7A5*, which encodes a tryptophan transporter, and *IDO1* was increased in the blood of infected rhesus and humans, supporting leukocytes as sources of kynurenine. Tryptophan has been found to be enriched in blood during cerebral malaria,^35^ although other studies identified reduced tryptophan abundance in the blood of humans with severe and uncomplicated *P. falciparum* malaria,^36^ in humans infected by *P. vivax*,^25,32^ and in a different controlled *P. cynomolgi* infection.^37^ These results suggest that the balance between tryptophan and kynurenine in blood is affected by leukocyte responses; however, the gut microbiome displays potential for tryptophan biosynthesis during *P. cynomolgi* infection^37^ and can impact host tryptophan metabolism. Recent findings suggest that IDO1-catabolized tryptophan-derived metabolites activate the aryl hydrocarbon receptor (AhR) and promote regulatory T cells in *P. vivax* infection,^38^ providing further insights into the functional roles of tryptophan metabolism in malaria.

The androgen and estrogen biosynthesis and metabolism pathway was enriched across all infections in rhesus and humans at the metabolite level, which suggests functional roles for these metabolites in malaria. The pathway has been previously implicated in metabolomics evaluation of *P. vivax* recurrence and treatment in an independent cohort.^39,40^ Most known for their role as sex hormones, these steroids present immunomodulatory functions and can be produced in the gonads and adrenal glands. Malaria promotes immunomodulation via steroids produced by adrenal glands such as cortisol and pregnenolone sulfate,^21,26^ which are critical for mice survival upon *Plasmodium* infection.^41^ Our results also highlight the conserved reaction between 3-methoxy-4-hydroxyphenylacetaldehyde and phenylacetaldehyde in *P. coatneyi* and human malaria. These results suggest the activity of enzymes such as aldehyde dehydrogenase or monoamine oxidase, which can also catalyze other reactions depending on the substrate. Genes encoding these enzymes were not differentially expressed in blood from rhesus or humans, suggesting that other compartments are sources for these metabolites.

Acute malaria induces lymphopenia in humans, and the reduction of T and B cells in the periphery of macaques likely reflected their migration to secondary lymphoid organs such as the spleen.^42^ Despite that, *P. coatneyi* induced a higher frequency of proliferating T lymphocytes that was also evident at the transcriptional level by gene modules involved in cell division, but not in *P. vivax* infection. KI67+ central memory CD4+ T cells also displayed an antigen-activated phenotype, as suggested by higher CD28 and PD-1 expression. In line with our findings, controlled *P. falciparum* infection in humans induces higher activation of CD4+ T cells compared to *P. vivax*.^10^ CD8+ T cell reductions were more pronounced in *P. cynomolgi* infection, suggesting a prominent role for CD8+ T cells during blood-stage infection as seen for *P. vivax*.^43^ Lymphocyte cell egress from lymphoid organs is controlled by sphingosine-1-phosphate (S1P);^28^ thus, reduced levels of plasma S1P during *P. cynomolgi* infection likely explain the retention of many T cells out of the peripheral blood. There are differences in cellular populations when compared to human malaria; however, it should be considered that the parasite cycle also differs significantly. Indeed, *P. falciparum* or *P. vivax* reaches peak parasitemia much faster in controlled human infections,^10,44^ characterizing the different biology of parasites and hosts.

Integration of different data types resulted in distinct molecular interaction networks, depending on the *Plasmodium* species. But it also demonstrated the redundancy of pathways that seem to be activated by specific species. Glycerophospholipid metabolism was associated with *P. cynomolgi* infection at both the transcriptional and metabolomic layers, suggesting this pathway can be important for leukocyte function only in this infection. However, network analysis suggests metabolites involved with glycerophospholipid metabolism are correlated with the activity of B cell, T cell, and DC subsets during *P. coatneyi* infection. Furthermore, the transcriptional activity of *P. falciparum* is correlated with host glycerophospholipid metabolism both at the transcriptional and metabolite layers,^45^ whereas peripheral *P. vivax* parasitemia is inversely associated with glycerophosphocholine and phosphocholine levels.^46^ Collectively, these studies point out critical roles for glycerophospholipid metabolism in leukocytes during malaria.

Even though infections were performed in a controlled environment, they occurred at different periods of time and in different animals, which can introduce technical and unknown confounding effects to the data. Sample sizes are also limitations inherent in the original study designs, whose intention was the follow-up of animals for over 100 days after the initial infection. Data from humans were obtained from children (transcriptomics) and adults (metabolomics). Therefore, pathways and molecules that were exclusively associated with one determined *Plasmodium* species cannot be distinguished from those significant due to the heterogeneity of each rhesus macaque and human cohort. Therefore, careful consideration is needed to ascertain which of the discussed molecules and processes can be associated with specific malaria parasites. Other limitations include metabolite annotations, whose identities were not confirmed using chemical standards, or the lack of functional validation that could prove a causal relationship between the pathways and molecules and the phenotypical observations. Nevertheless, our study demonstrates that infection with distinct *Plasmodium* species mobilizes diverse cell subsets and molecular pathways. Targeting conserved or species-specific features may aid in the development of host-directed and personalized malaria therapies and vaccines.

## Methods

### Data Compilation and Processing

The data sets used in this study were acquired by the Malaria Host–Pathogen Interaction Center (MaHPIC) and deposited in specialized databases. The data sets were collected during MaHPIĆs experiments E03 (*P. coatneyi* infection)^17^ and E04 (*P. cynomolgi* infection).^20^ We used data collected before infection (baseline) or at peak parasitemia (acute infection). We discarded other time points because the regimen of treatment after peak parasitemia differed between infections and thus could influence both immune and metabolic responses. The experimental designs were similar between experiments before infection and at the peak of parasitemia, differing mostly by the initial parasite inoculum, which was approximately 100 sporozoites of *P. coatneyi* Hackeri strain^17^ and 2000 sporozoites of *P. cynomolgi* B strain.^20^ Cell blood counts and parasitological data were retrieved from the Clinical Malaria Core data at the MaHPIC data repository section in the PlasmoDB database (https://plasmodb.org/plasmo/app/static-content/PlasmoDB/mahpic.html). Preprocessed flow cytometry data were downloaded from the ImmPort database under accession ID SDY1411 for experiment E03 and accession ID SDY1015 for experiment E04. *M. mulatta* genome-aligned read count tables resulting from RNA sequencing (RNaseq) data were found in the Gene Expression Omnibus (GEO) database under accession ID GSE103259 for experiment E03 and accession ID GSE10350741 for experiment E04. RNaseq data sets from whole blood of children infected with *P. vivax* and uninfected controls were also retrieved from GEO under the accession ID GSE144792,^47^ whereas data for *P. falciparum* infection is deposited at the European Nucleotide Archive (ENA) under the accession ID PRJEB45911.^48^ Untargeted metabolomics data, acquired with liquid chromatography–mass spectrometry (LC–MS) and extracted with apLCMS,^49^ were downloaded from the MetabolomicsWorkbench database under the accession ID ST000599 for experiment E03 and accession ID ST000515 for experiment E04. Untargeted metabolomics data from adults infected with *P. falciparum* or *P. vivax* were extracted from the MetaboLights database under the accession ID MTBLS664.

### Data Filtering, Normalization, and Transformation

Parasitemia, CBC data, and flow cytometry data (absolute cell number and median fluorescence intensity) were log2-transformed before statistical analysis. RNaseq data were filtered and normalized to log counts per million reads (logCPM) with edgeR v4.2.2. For untargeted metabolomics data, we used the package MSprep v1.14.0 to average replicates, filter peaks present in at least 60% of all samples, impute missing values using half of the minimum intensity for a determined *m*/*z* peak, and transform data to the base 2 logarithmic scale. An *m*/*z* peak is defined by *m*/*z*, retention time, and associated intensity.

### Statistics and Functional Analyses

The Shapiro–Wilk normality test was used to analyze data distribution of CBC data. Comparison of parasitemia and CBC data between experiments was performed with the Mann–Whitney test using baseline-subtracted values (log2 fold-change) to remove individual confounding effects at acute infection. Paired data from the same animal at different time points was analyzed with a paired *t*-test. We used the limma package v3.60.6 to identify significant differences in flow cytometry, RNaseq, and LC–MS data, controlling false discovery rate (FDR) with the Benjamini–Hochberg procedure.

Gene ontology (GO) analysis for transcriptomics data was performed with ToppGene suite,^50^ and gene set enrichment analysis (GSEA) was performed using the Blood Transcription Module (BTM) plus as gene sets.^23^ Metabolite annotations and predictions of the metabolic pathway from untargeted metabolomics data were calculated with mummichog v2.7.0.^27^ Volcano and bubble plots were generated with the ggplot2 package v3.5.1. Networks were visualized with Cytoscape v3.10.0.

Integration of orthogonal data was performed using a multifactorial, multiscale network response approach.^25,29,51^ Cell frequencies, transcriptomics, and metabolomics data from acute infection were baseline-subtracted to remove individual confounding effects. Transcriptomics data dimension was then reduced to 273 BTMs for experiment E03 and 275 BTMs for experiment E04. Next, BTMs, cell frequencies, and metabolomics data were grouped into clusters with unsupervised hierarchical clustering and Pearson correlation as metrics. For metabolomics data, we also used retention time to group different ions deriving from the same metabolite within the same cluster.^25^ To identify associations between clusters of the same data type or different data, we calculated partial least-squares (PLS) correlations, and permutation was used to evaluate the significance of such associations (*p*-value), which connect the nodes in the network. Nodes in the resulting networks are subnetworks linking unique features of the same data type, thus characterizing the hierarchical topology of the multimodal network.

## Supplementary Material



## Notes

Transcriptomic analysis of rhesus macaques infected with *P. coatneyi* or *P. cynomolgi*; gene set enrichment analysis of rhesus macaque and human transcriptomics data; metabolomics analysis of rhesus macaques infected with *P. coatneyi* or *P. cynomolgi* or humans infected with *P. falciparum* or *P. vivax*; and members of BTM clusters 11 and 7 relative to Figure 5A,B (PDF)
