# Supplementary material for: Systems Biological Analysis of Immune-Metabolic Host Responses to Distinct Malaria Parasites
Source: ACS Omega. 2025 Oct 13;10(41):49061–73. doi: 10.1021/acsomega.5c07785 (PMC12573300; doi:10.1021/acsomega.5c07785)
Supplement: Supplementary file 1 [file ao5c07785_si_001.pdf]

Systems biological analysis of immune-metabolic host responses to distinct malaria parasites

Davi Vinícius Lima[1], Tiago Paiva Guimarães[1], Anne Cristine Gomes Almeida[1,2], Arthur Jesuz Teixeira[3], Wuelton Marcelo Monteiro[2,4], Gisely Cardoso Melo[2,4], Luiz Gustavo Gardinassi[1,3*]

1 – Programa de Pós-Graduação em Medicina Tropical e Saúde Pública, Instituto de Patologia Tropical e Saúde Pública, Universidade Federal de Goiás, 74605-050, Goiânia - GO, Brazil

2 – Gerência de Malária, Fundação de Medicina Tropical Doutor Heitor Vieira Dourado, 69040-000, Manaus - AM, Brazil

3 – Departamento de Enfermagem Materno-Infantil e Saúde Pública, Escola de Enfermagem de Ribeirão Preto, Universidade de São Paulo, 14040-902, Ribeirão Preto – SP, Brazil

4 – Escola Superior de Ciências da Saúde, Universidade do Estado do Amazonas, 69065-001, Manaus – AM, Brazil

Correspondence: Luiz Gustavo Gardinassi. Departamento de Enfermagem Materno-Infantil e Saúde Pública, Escola de Enfermagem de Ribeirão Preto, Universidade de São Paulo, 14040-902, Ribeirão Preto – SP, Brazil. Telephone: +55 16 3315-3395. E-mail address: gardinassi@eerp.usp.br

Supporting information

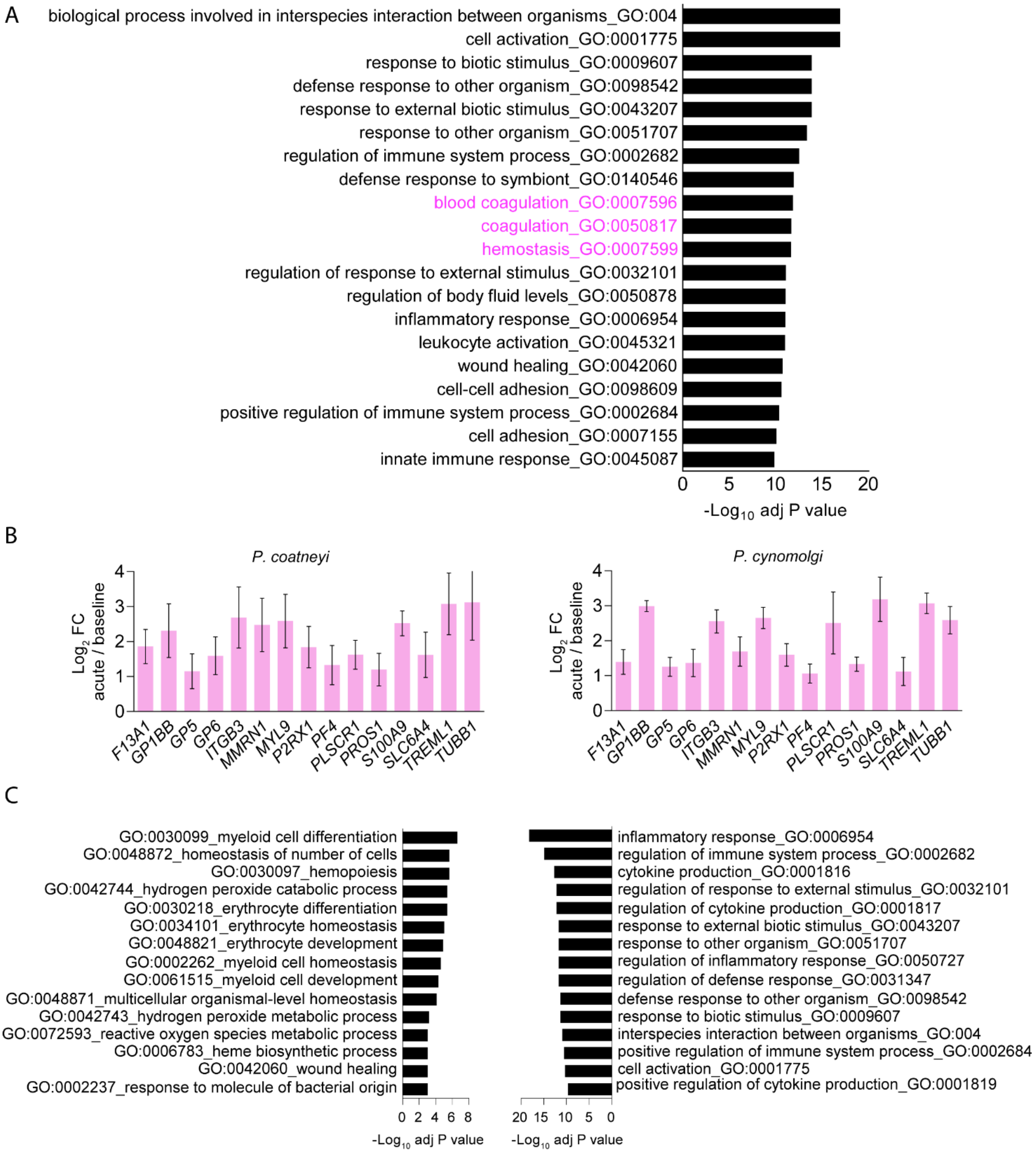

Figure S1 – Conserved and species-specific transcriptional responses of rhesus macaque to *Plasmodium* infection. (A) Enrichment in biological processes from gene ontology (GO) for conserved DEGs between infections with *P. coatneyi* or *P. cynomolgi*. (B) Expression of genes involved in coagulation and homeostasis at acute infection normalized by baseline levels. (C) Enrichment in biological processes from GO for exclusive DEGs for infections with *P. coatneyi* (left) or *P. cynomolgi* (right).

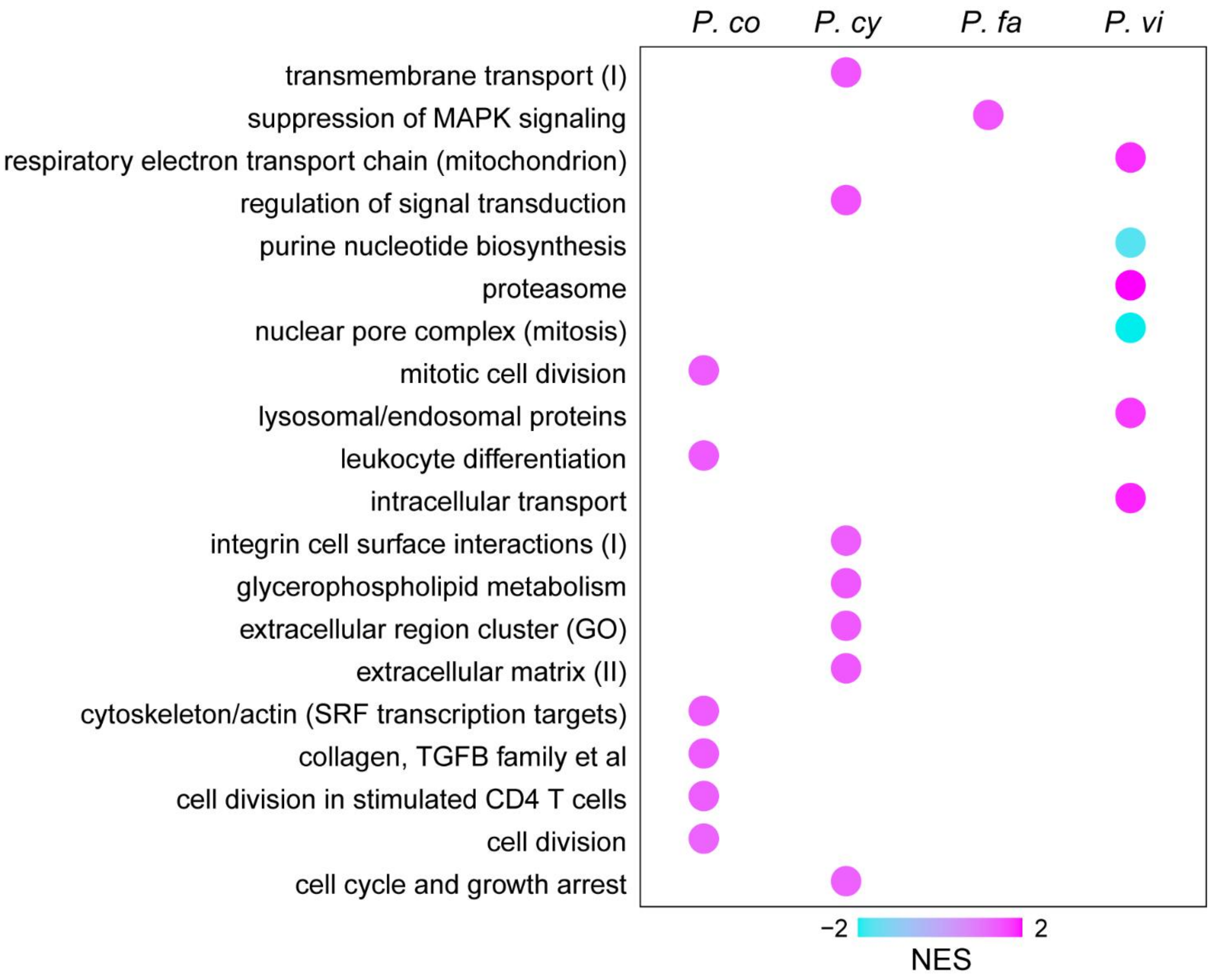

Figure S2 – Species-specific BTM responses of rhesus macaque and humans to *Plasmodium* infection. Gene set enrichment analyses using the Blood Transcription Modules framework of simian and human *Plasmodium* infections.

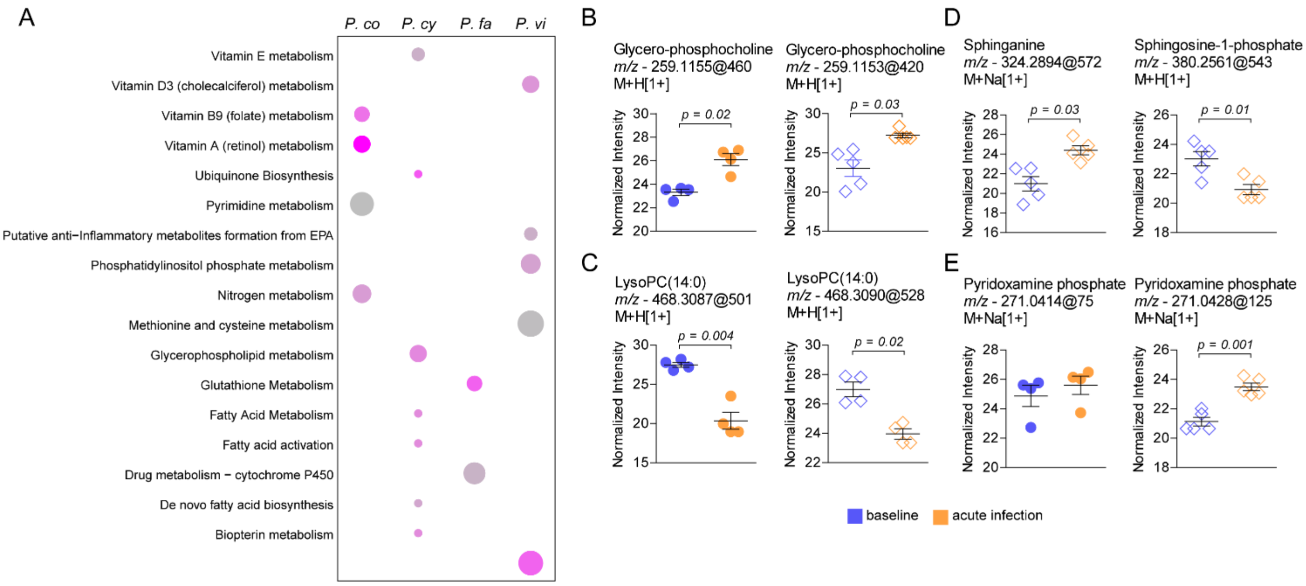

Figure S3 – Species-specific metabolic responses of rhesus macaques and humans to *Plasmodium* infection. (A) Metabolic pathway prediction analysis in rhesus and humans infected with different *Plasmodium*. (B - E) Normalized abundance of glycerol-phosphocholine (B), lysoPC(14:0) (C), sphinganine and sphingosine-1-phosphate (D) and pyridoxamine phosphate (E).

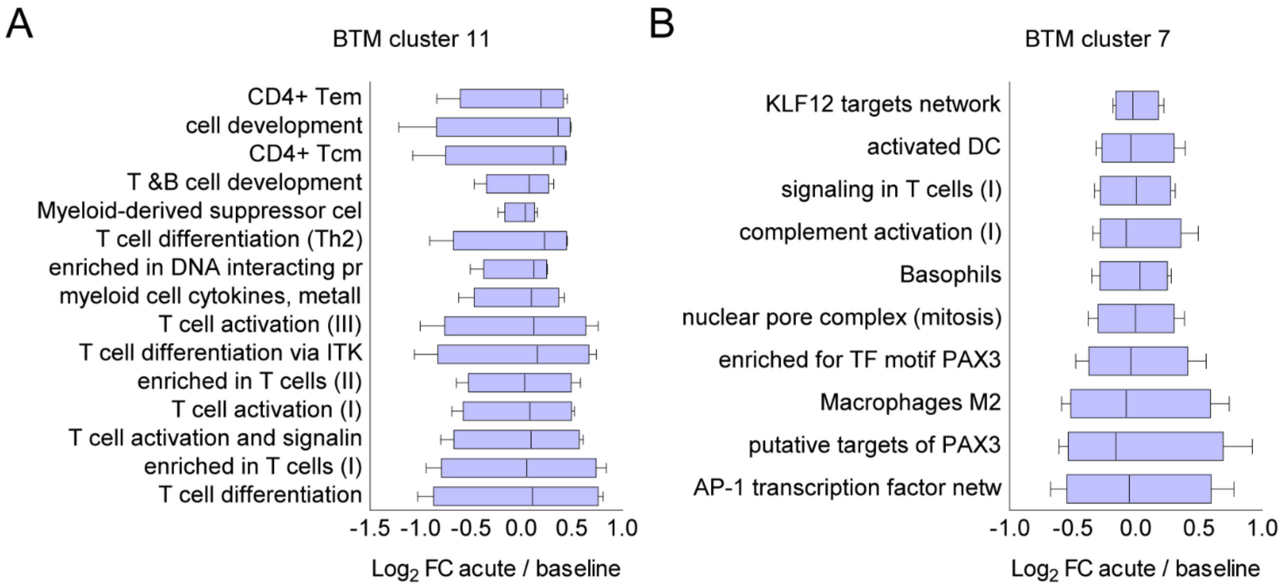

Figure S4 – BTMs composing clusters associated with cell cluster C.2. (A) BTMs composing BTM cluster B.11. (B) BTMs composing BTM cluster B.7. Relative to figure 5A-B
